# Scoping Review of Pediatric Early Warning Systems (PEWS) in Resource-Limited and Humanitarian Settings

**DOI:** 10.3389/fped.2018.00410

**Published:** 2019-01-08

**Authors:** Stephanie R. Brown, Daniel Martinez Garcia, Asya Agulnik

**Affiliations:** ^1^Seattle Children's Hospital, University of Washington, Seattle, WA, United States; ^2^Women & Child Health & Nutrition Unit, Medical Department, Médecins Sans Frontières (MSF), Operational Center Geneva, Geneva, Switzerland; ^3^Department of Global Pediatric Medicine, St. Jude Children's Research Hospital, Memphis, TN, United States

**Keywords:** pediatric early warning system, humanitarian pediatrics, resource-limited settings, quality of care, hospital mortality

## Abstract

Pediatric Early Warning Systems (PEWS) aim to identify hospitalized children at increased risk of deterioration by assigning a score based on vital signs and clinical status and guiding interventions using a response algorithm to improve outcomes. When implemented with quality improvement methodology, these systems have been shown to be effective in high-resource settings and have the potential to improve the care of children in humanitarian and resource-limited settings (RLS). The purpose of this review is to summarize the current evidence for use of PEWS in RLS and identify areas for further research. A review of the current PEWS literature in RLS was performed using Web of Science, PubMed, Scopus, Cumulative Index of Nursing and Allied Health Literature (CINAHL), EMBASE, Portal Regional da BVS, and TRIP Database. While there is limited research available on this topic, eight studies on the use of PEWS, or a PEWS score in a pediatric population in low- or middle-income countries were identified. Two studies assessed the clinical effect of implementation of PEWS; one reported a reduction in clinical deterioration events and the other a reduction in mortality. The remaining studies assessed the association of a PEWS score with signs of clinical deterioration or mortality without a response algorithm. Further research on the impact of PEWS implementation on inpatient care and outcomes in RLS is needed.

## Introduction

A variety of Pediatric Early Warning Systems (PEWS) have been proposed by multiple groups working in hospitals worldwide (1–4). The system consists of two components; the scoring tool, which is calculated at regular intervals during hospital admission and a response algorithm with interventions and/or provider assessments triggered based on the PEWS score. PEWS scoring tools typically incorporate clinical information such as vital signs, neurologic status, work of breathing, and perfusion. A broad range of systems are currently in use with variable accuracy in identifying deterioration (5). PEWS response algorithms also vary; in some cases a high PEWS score leads to evaluation by a more senior nurse or physician and in others it triggers a rapid response team activation, typically consisting of clinicians with critical care training, or Intensive Care Unit (ICU) consultation (6, 7). Successful implementation of PEWS requires a quality improvement approach and adjustments often need to be made to the system to adapt the score and algorithm for a particular clinical context or patient population. Multiple studies have retrospectively or prospectively validated PEWS in high-resource settings, with Area Under the Receiver Operating Characteristic curve (AUROC) of different scoring systems ranging from 0.73 to 0.91 (1, 3, 8–10). While the reliability and validity of PEWS has been demonstrated, there is conflicting evidence that the use of PEWS impacts patient outcomes such as frequency of cardiac or respiratory arrest or hospital mortality in high-resource settings (11).

The term resource limited setting (RLS) describes a wide spectrum of clinical contexts, typically found within low- and middle-income countries, where there is inadequate access to necessary supplies and personnel. There is relatively little research conducted in RLS and applying research findings between settings can be difficult, since the patient population and capacity of each context can vary substantially. This problem is amplified in one specific type of RLS, humanitarian settings, where conducting research can be nearly impossible. Humanitarian settings are characterized by extremely limited personnel and equipment and provide care for populations affected by conflict or natural disaster. In these contexts, the local capacity is overwhelmed and non-governmental organizations (NGOs) provide additional resources where possible. While humanitarian settings can be extremely difficult to work in, it is critical to make every attempt to provide high-level-care regardless of context capacity. To achieve this, standards of care need to be adapted from high-resource and other RLS to meet the special needs of humanitarian settings. It is also crucial to conduct original research, wherever possible, in humanitarian settings to build a body of literature specific to this context.

Hospitals in humanitarian and RLS face specific challenges, making early identification of children at risk for critical deterioration more difficult. The demands on nurses can be extraordinary, with nurse-to-patient ratios as high as 1:50 during the day and over 1:100 at night (12), making it difficult to systematically collect vital signs and perform thorough clinical assessments. It has also been noted that many nurses caring for children in RLS may not have extensive pediatric experience, which makes depending on clinical judgment very difficult (13). These challenges are compounded by the presence of relatively few doctors responsible for large numbers of patients, making it critically important that deteriorating children are efficiently identified and brought to the attention of these providers. Implementation of PEWS may allow staff to identify clinical changes early and intervene before a serious deterioration event, potentially preventing the need for intensive therapies, or transfer to another facility. Prevention of clinical deterioration is especially valuable in humanitarian and RLS, where the personnel and equipment required to resuscitate a critically ill child may be limited or not available. Despite these potential benefits, there have been few studies of PEWS implementation in humanitarian and RLS. The purpose of this scoping review is to identify and describe the current literature on PEWS use and impact in RLS and identify areas for further research.

## Methods

Articles for review were obtained by searching Web of Science, PubMed, Scopus, Cumulative Index of Nursing, and Allied Health Literature (CINAHL), EMBASE, Portal Regional da BVS, and TRIP Database for all entries from database inception to November 2017 with the assistance of a librarian scientist. The databases were searched using key words including early warning system, rapid response, pediatric, and severity of illness. The search also included terms such as resource-limited, low- and middle-income countries, and the names of low- and middle-income countries (LMICs). For full details of the search strategy please see Appendix 1. Articles were included for review if they were written in English or Spanish and studied implementation of PEWS or use of a PEWS score in a pediatric population within a RLS. For the purposes of this review studies were considered from RLS if they self-identified as RLS or were conducted in an LMIC.

**Figure 1:**
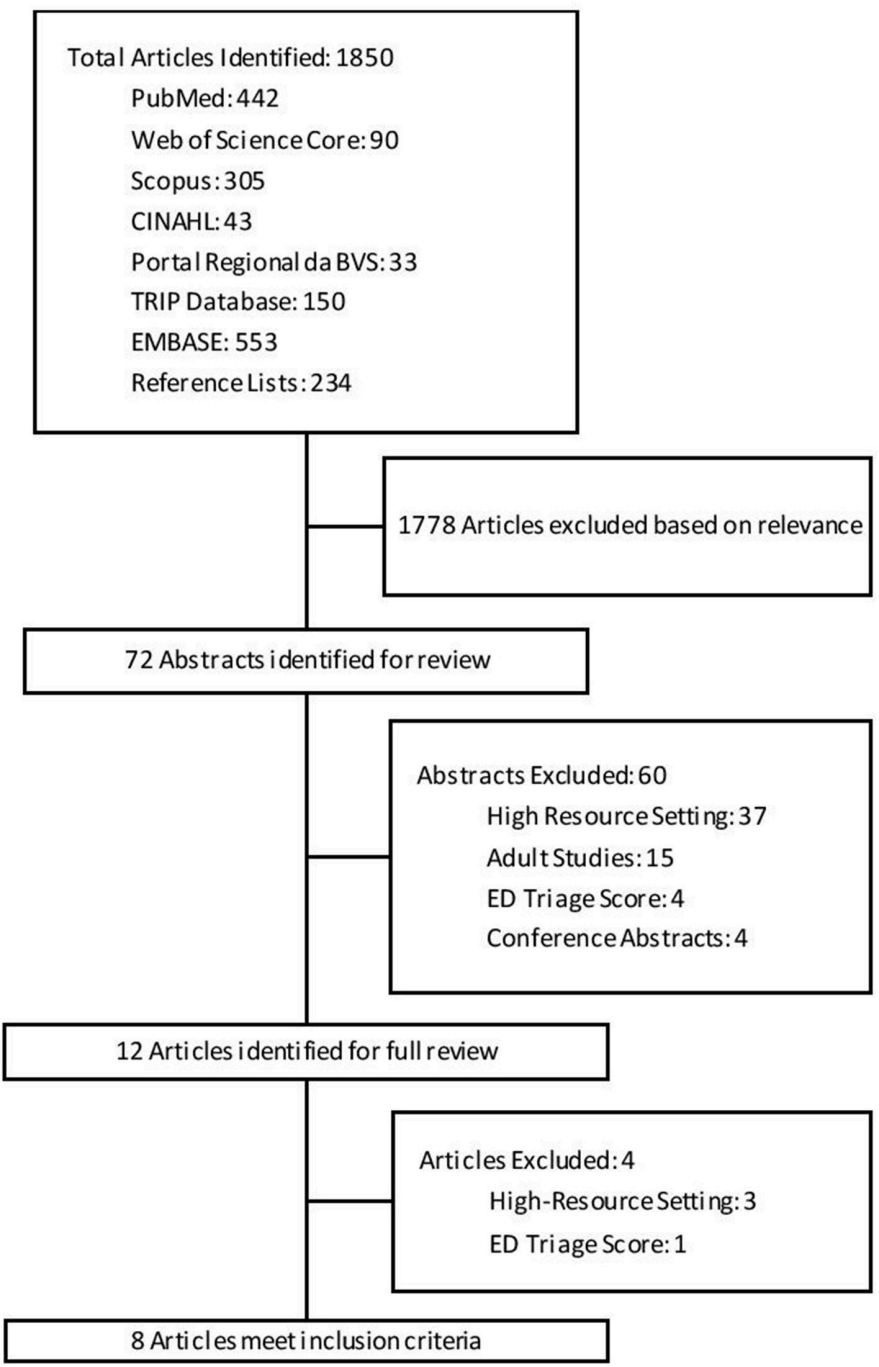
Flowchart of search results and article screening process.

This search identified 1,850 articles, from which 72 were found to be potentially relevant to this study. Review of the abstracts identified 8 articles meeting the inclusion criteria for this review, see Figure 1 for details of search results. The reference lists of included articles did not identify any further articles meeting inclusion criteria.

Information was extracted from the included studies regarding the setting, population, study design, objectives, PEWS version used and key results. For each PEWS scoring tool, details of the objective and subjective data used to calculate each score were noted as well as whether age adjustment or aggregate weighting was used. Additionally, it was noted whether calculation of the score required any medical equipment, such as a pulse oximeter, sphygmomanometer, or laboratory test.

## Results

### Study Characteristics

The literature search identified eight publications by five research groups meeting all inclusion criteria. The characteristics of these studies can be found in Table 1 (12–19). The studies took place in Malawi, Guatemala, Brazil, Thailand, Uganda, Tanzania, and Kenya. Four of the studies took place in large referral hospitals, three of which had intensive care or high-dependency units, including one in a pediatric oncology hospital (12–15, 18, 19). The fifth study took place in a combination of large referral centers and small district hospitals; no studies from humanitarian settings were identified (15). There were two studies that utilized PEWS with a response algorithm and one study described the translation and assessment of diagnostic accuracy without a response algorithm or evaluating impact of implementation (12–14, 17–19). The ability of a PEWS score to predict inpatient disposition for patients in the emergency room was described in one study (15). Lastly, one study described the development of a severity of illness score and used a PEWS score as one of the comparative measures (16).

**Table 1:** Characteristics of studies of pediatric early warning systems in resource limited settings.

**References**	**Country**	**Setting**	**No. of subjects**	**Age range**	**System**	**Study design**	**Purpose**	**Trigger & response**	**Key results**
Olson et al. (12)	Malawi	Large referral hospital with high dependency unit	54 Cases; 161 Controls	< 15 years old	Inpatient Triage, Assessment and Treatment (ITAT)	Prospective nested case-control study	Develop ITAT score to identify clinical deterioration in inpatients and prompt physician evaluation	ITAT score >3 triggers physician assessment	ITAT score >3: OR for death 4.8, predicts mortality with AUROC 0.76
Olson et al. (14)	Malawi	Large referral hospital with high dependency unit	1,642 Assessments	< 15 years old	Inpatient Triage, Assessment and Treatment (ITAT)	Prospective quality improvement project	Improve pediatric inpatient surveillance by provision of vital sign equipment implementation of vital sign assistant program	ITAT score >3 triggers physician assessment	Vital signs assistants associated with increased frequency and accuracy of ITAT scores & increased clinician notiifcations; Mortality reduced from 9.3% to 5.7%
Chaiyakusil and Pandee (15)	Thailand	Emergency Department in tertiary university hospital with ICU	1,136	< 15 years old	Pediatric Early Warning Score (PEWS)	Prospective descriptive study	Validation of PEWS on arrival in Emergency Department to predict inpatient admission disposition	None	Predicts Admission: AUROC 0.73, PEWS 1 or above, Sensitivity 78%, Specificity 59.6%; Predicts ICU admission: AUC 0.98, PEWS 3 or above, Sensitivity 100%; Specificity 90.5%
George et al. (16)	Uganda, Tanzania & Kenya	Mix of 6 large and small hospitals	3,125^*^; 1,053^**^; 5,098^**^	2 months - 12 years	Bedside PEWS	Retrospective data used to develop and validate PET score	Develop an easy to use, bedside score for risk of mortality based on clinical signs	None	PET score associated with mortality w/in 48 hrs w AUROC 0.77–0.86; Predicts mortality better than PEWS, PRISMIII & Aquamat
Miranda et al. (17)	Brazil	Pediatric referral hospital without ICU	271	0–10 years	Brighton PEWS (BPEWS)	Integrative Review	Literature review, translation and adaptation of BPEWS for Brazilian Portuguese and Pilot test	None	BPEWS was successfully translated and adapted for Brazilian Portuguese and in the pilot study, 26.6% of children were considered at risk of clinical deterioration based on PEWS score >2
Miranda et al. (18)	Brazil	Pediatric referral hospital without ICU	271	0–10 years	Brighton PEWS adapted for Brazilian Portuguese (BPEWS-Br)	Prospective evaluation of diagnostic acuracy	Evaluation of diagnostic accuracy of BPEWS-Br compared to provder assessment of clinical deterioration	None	BPEWS-Br associated with clinical signs of deterioration, AUROC 0.919; BPEWS-Br score >2, sensitivity 73.9% and specificity 95.5%
Agulnik et al. (13)	Guatemala	Pediatric oncology hospital with ICU	129 Cases; 129 Controls	< 18 years	Pediatric Early Warning Score (PEWS)	Retrospective matched case-control study	Validation of the ability of PEWS to predict the need for unplanned ICU transfer	PEWS algorithm followed, high PEWS prompted physician and/or ICU evaluation	PEWS correlated with unplanned ICU transfer: AUROC 0.94; PEWS >3, sensitivity 88% and specificity 93%; Higher PEWS prior to PICU transfer associated with increased morbidity & mortality, higher PIM2 and increased support requirements in ICU
Agulnik et al. (19)	Guatemala	Pediatric oncology hospital with ICU	287	< 18 years	Pediatric Early Warning Score (PEWS)	Retrospective cohort study the year before and year after implementation of PEWS.	Describe the effect of implementation of a PEWS system on the frequency of clinical deterioration events	PEWS algorithm followed, high PEWS prompted physician and/or ICU evaluation	Abnormal PEWS in 93% of unplanned PICU transfer; Reduced frequency of clinical deterioration events, severe sepsis, septic shock and organ dysfunction; Reduced ICU utilization; No change in mortality

* Development data set;

** *Validation data set*.

The components of each PEWS scoring tool utilized can be found in Table 2 and further details of the scoring tools can be found in Appendix 2. There was some variation between the scoring tools used in each study, although all used an aggregate weighted system. Only one score did not include adjustments for age or require any additional equipment and another did not require any subjective data to be collected (12, 14, 16). For the remainder of the scoring tools, calculating the score required some aspects of a physical exam to assess neurologic status, perfusion, and respiratory effort.

**Table 2:** Components utilized in calculation of score.

		**Objective data**								**Subjective data**				
**References**	**System**	**Age adjusted**	**Aggregate weighted score**	**Temperature**	**Heart rate**	**Respiratory rate**	**Blood pressure**	**Oxygen saturation**	**Oxygen support**	**Neurologic**	**Respiratory**	**Perfusion**	**Other**	**Equipment required**
Olson et al. (12, 14)	Inpatient Triage, Assessment and Treatment (ITAT)	✓	✓	✓	✓	✓		✓						✓
Chaiyakusil and Pandee (15)	Pediatric Early Warning Score (PEWS)	✓	✓	✓	✓	✓	✓	✓	✓	✓	✓	✓	✓	✓
George et al. (16)	Bedside PEWS	✓	✓	✓	✓	✓	✓	✓	✓	✓	✓	✓	✓	✓
Miranda et al. (17)	Brighton PEWS adapted for Brazilian Portuguese (BPEWS-Br)	✓	✓		✓	✓			✓	✓	✓	✓	✓	✓
Agulnik et al. (19, 20)	Pediatric Early Warning Score (PEWS)	✓	✓		✓	✓		✓	✓	✓	✓	✓	✓	✓

*✓: component utilized*.

### Reliability

Three studies found that PEWS scores could be accurately and reliably calculated by clinical staff in RLS (13, 15, 19). One study had all assessments calculated by a single nurse and the last study calculated scores based only on retrospective data from a previous study (16, 18).

### Validity

Overall, PEWS scores accurately identified children with a higher severity of illness in these settings. In their validation study, Agulnik et al. found that an elevated PEWS score correlated with unplanned ICU transfer, with an AUROC of 0.94, with sensitivity of 88%, and specificity of 93% for a PEWS score of 5. Furthermore, they found that higher PEWS scores prior to transfer to the ICU were associated with increased morbidity and mortality (13). Olson et al. validated their inpatient triage, assessment and treatment (ITAT) score and determined that it was associated with mortality within the next 48 h with an AUROC of 0.76, sensitivity of 44% and specificity of 86% (14). Miranda et al. found that an elevated PEWS score was associated with clinical signs of deterioration with an AUROC of 0.92, sensitivity of 74%, and specificity of 96% (18). An elevated PEWS score assessed in the Emergency Department of a university hospital was associated with admission to the hospital with an AUROC of 0.73, sensitivity of 78%, and specificity of 60%. Higher PEWS scores were also associated with ICU admission, with an AUROC of 0.98, sensitivity of 100% and specificity of 91% (15). Finally, George et al. calculated PEWS scores for all patients in three data sets and found that their Pediatric Emergency Triage (PET) severity of illness score was more strongly associated with mortality within 48 h than PEWS score with an AUROC of 0.77–0.86 vs. 0.64–0.74 respectively (16).

### Clinical Impact

After implementation of PEWS, Agulnik et al. reported a reduction in the frequency of deterioration events from 9.3 to 6.5 per 1,000 inpatient days (19). They also report a reduction in the number of ICU transfers required for septic shock and in rates of organ dysfunction upon admission to the ICU. Additionally, there was reduced ICU utilization for unplanned transfers despite an overall increase in hospital admissions. Olson et al. implemented their ITAT system initially within their existing hospital structure and despite training nurses to use ITAT, the rate of assessment decreased from 0.67 to 0.61 per patient per hospitalization (14). The introduction of additional staff trained to collect vital signs, called vital sign assistants, was associated with an increase in frequency of assessments to 2.44 per patient per hospitalization, more frequent provider notifications and a reduction in mortality from 9.3 to 5.7%. The George, Miranda and Chaiyakusul groups did not implement PEWS in their hospitals, so clinical effectiveness could not be determined.

## Discussion

Our scoping review identified limited data on the use of PEWS in RLS and no work describing PEWS in humanitarian settings. The available evidence, however, suggests that successful implementation of PEWS is possible in these settings, and may be associated with a reduction in clinical deterioration events and hospital mortality (14, 19). One of the challenges of interpreting the small amount of existing literature on the use of PEWS in RLS is wide variability in how PEWS is utilized. In this review, we found that early warning scores are frequently used as a severity of illness measure without an accompanying response algorithm, focusing only on the score itself rather than the entire early warning system. The available research from high-resource settings suggests that an improvement in outcomes requires implementation of the scoring tool and algorithm together (11, 21). PEWS scoring tools are not intended as severity of illness tools to predict risk of mortality, nor are they validated for this purpose.

Another challenge facing those working in humanitarian and RLS is the heterogeneity of clinical contexts, which limits applicability between various settings. The term “resource-limited” describes an enormous variety of clinical contexts, capacities, and patient populations. Clinical settings within LMICs are generally considered RLS, however tertiary centers frequently have substantially more resources than other settings within the same country. In the current study, we considered studies to be from RLS if they were set in an LMIC or were identified as RLS by the authors. All of the studies included in this review were set in LMICs; five of eight self-identified as resource limited, and two of the remaining three described limited availability of equipment, monitoring, and staff. Understandably, the majority of research in RLS comes from the relatively high-resource university hospitals in more stable political contexts. This makes the findings from such research less generalizable to other RLS, especially humanitarian contexts. While there are unique challenges to conducting research in humanitarian settings, work in this area is urgently needed to improve hospital quality of care for children regardless of their circumstances.

While the body of literature on PEWS in high-resource settings continues to grow, much of this research is difficult to translate to resource-limited contexts. The constraints of RLS present challenges to PEWS implementation. First, it is difficult to use a system relying on the collection of frequent vital signs and clinical assessments when nurse to patient ratios are as low as 1:50. This reality was illustrated by Olson et al. when they were successful in implementation of their ITAT system only with the introduction of vital sign assistants (14). This is further complicated by variable pediatric experience among staff, making clinical evaluations of mental status and respiratory effort potentially unreliable. Additionally, equipment required to obtain vital signs such as a pulse oximeter or sphygmomanometer, as well as laboratory facilities, may not be available in all settings, making some more complicated scoring tools impossible to use. Another challenge is that many systems include assessment by a critical care physician or transfer to the ICU as part of the response algorithm. This is not possible in settings that do not have an ICU or high dependency area and requires adjustment of the algorithm to match the capacity of the local context. Finally, while the aim of PEWS implementation in high-resource settings is to prevent cardiopulmonary arrests on the floor and transfer patients to the ICU at an earlier stage of illness, the aim in many humanitarian and RLS may be to prevent critical illness where ICU resources are limited, thus reducing mortality. The broad range of clinical contexts found in humanitarian and RLS demands a PEWS scoring tool and algorithm that can be adapted to the patient population and capacity of the setting.

There are significant potential benefits from PEWS implementation in humanitarian and RLS. PEWS may aid in the triage of large numbers of hospitalized patients and help staff identify those who require immediate attention without the need for robust pediatric experience. The use of a standardized assessment tool may also improve the clarity and efficiency of communication between nurses and doctors. PEWS implementation, through the reduction of clinical deterioration events, can reduce overall personnel and equipment costs of hospital care, as has been suggested through cost-benefit analysis in high- and low-resource settings (19, 20, 22). Studies from high-resource settings have failed to show a decrease in hospital mortality following PEWS implementation, likely due to low baseline hospital mortality and an existing thorough baseline monitoring of hospitalized patients in these settings (11). As such, the effectiveness of PEWS is frequently assessed using surrogate measures of morbidity and mortality such as reduction in critical deterioration events or unplanned transfer to the ICU (23). However, baseline pediatric inpatient mortality in humanitarian and RLS is much higher than in high-resource settings. A study by van den Boogaard et al. found that among eight hospitals in Africa supported by Médecins Sans Frontières, pediatric inpatient mortality rates ranged from 3 to 9% (24). Similarly, groups working in Uganda and Tanzania reported pediatric inpatient mortality rates of 2.7–3.5 and 7%, respectively (25, 26). In RLS, where inpatient mortality rates are much higher and capacity for patient monitoring is reduced, there is evidence that implementation of PEWS may reduce inpatient mortality (14).

While there are many possible benefits to PEWS implementation, there are also some potential drawbacks. For example, the time required to obtain vital signs, calculate the PEWS scores and respond to elevated scores, may dilute any benefit of the system if the tool is overly sensitive with a high rate of false positives. One way to limit this risk is to develop PEWS systems that reduce false positives by ensuring that the number needed to evaluate is low in these settings. Dean and colleagues demonstrated this in their study, which found that while their PEWS had an AUROC of 0.91, the number needed to evaluate ranged from 4.4 (PEWS ≥ 5) to 20.5 (PEWS ≥ 3) (27). This potentially high number of false-positives is problematic in any setting but could be devastating in a humanitarian and RLS. Fortunately, the number needed to evaluate tends to be low in high risk populations, with high baseline rates of clinical deterioration, as are often found in humanitarian and RLS. Another potential problem is the misinterpretation and use of the PEWS score as a diagnostic rather than a screening tool. This is a common misunderstanding and could lead to patients with clinical deterioration being inappropriately labeled stable based on a low PEWS score. It is critical to implement PEWS with appropriate education and to reinforce that parental, nursing, or physician concern should not be ignored in the presence of a low PEWS result. Successful implementation of PEWS requires a quality improvement approach and robust implementation methodology to address these potential challenges.

To our knowledge, this is the first comprehensive literature review on PEWS in RLS and humanitarian settings. The current study, however, has several limitations. Our literature search only identified 8 publications describing PEWS in RLS, and none from humanitarian settings. Based on our comprehensive search strategy conducted with assistance from a librarian scientist, we are confident that this represents a true reflection of the English and Spanish literature available on this subject. We believe this represents a gap in the existing literature and highlights the need for further study on this important topic. One challenge identified by our review is the heterogeneity of RLS identified in the available PEWS literature, ranging from small district hospitals to subspecialty referral centers, making generalizing their results to all RLS difficult. The findings of the available studies, however, suggest that PEWS may be particularly efficacious in environments, like RLS, with high inpatient mortality and frequent clinical deterioration. Additionally, only three of the studies identified utilized a scoring tool and response algorithm together. The PEWS scoring tool is not designed to be a measure of severity and is not effective on its own, more research is needed on the system as a whole. However, the broad utilization of the PEWS score in multiple clinical settings to identify children who may be at risk for clinical deterioration is an important first step.

## Conclusions

There is little existing literature to guide the use of PEWS in RLS and no literature on their use in humanitarian settings. The studies available have demonstrated the potential to reduce mortality while also reducing resource utilization in these settings. It is critical that further research is conducted to develop an adaptable framework of PEWS that can be adjusted to local resources and context. The operational impact of implementation of PEWS on the rate of clinical deterioration, length of stay, hospital morbidity, mortality, resource utilization, interdisciplinary communication, staff and patient satisfaction, and overall cost-effectiveness must be studied in humanitarian and RLS.

## Author Contributions

SB, DM, and AA contributed to the conception and design of the study. SB conducted the search of the literature with the assistance of a librarian scientist and wrote the first draft of the manuscript. All authors contributed to the revision of the manuscript and approved the submitted version.

### Conflict of Interest Statement

The authors declare that the research was conducted in the absence of any commercial or financial relationships that could be construed as a potential conflict of interest.
