# Supplementary material for: Scoping Review of Pediatric Early Warning Systems (PEWS) in Resource-Limited and Humanitarian Settings
Source: Front Pediatr. 2019 Jan 8;6:410. doi: 10.3389/fped.2018.00410 (PMC6331420; doi:10.3389/fped.2018.00410)
Supplement: Supplementary file 1 [file Table_1.DOCX]

Appendix 1. Details of Search Strategy

**Databases Searched:**

Web of Science

PubMed

Scopus

Cumulative Index of Nursing and Allied Health Literature (CINAHL)

EMBASE

Portal Regional da BVS and TRIP Database

**Key Words:**

PEWS

early warning

itat

critical deterioration

deteriorat*

severity of illness index

severity of illness

trigger system

alert criteria

pediatric

child

children

childhood

infant

infancy

adolescent

developing country

developing world

resource limited

resource poor

resource limiting

resource constrained

low and middle income countries

LMIC

asia

africa

south america

oceania

latin america

global health

Albania

Algeria

American Samoa

Samoa

Angola Azerbaijan

Belarus

Belize

Bosnia and Herzegovina

Bosnia

Herzegovina

Botswana

Brazil

Bulgaria

China

Colombia

Costa Rica

Cuba

Dominica

Dominican Republic

Ecuador

Fiji

Gabon

Grenada

Islamic Republic of Iran

Iran

Iraq

Jamaica

Jordan

Kazakhstan

Lebanon

Libya

Republic of Macedonia

Macedonia

Malaysia

Maldives

Marshall Islands

Mauritius

Mexico

Mongolia

Montenegro

Namibia

Palau

Panama

Paraguay

Peru

Romania

Serbia

South Africa

St. Lucia

Saint Lucia

St. Vincent and the Grenadine

Saint Vincent

Grenadines

Suriname

Thailand

Tonga

Tunisia

Turkey

Turkmenistan

Tuvalu

Armenia

Bangladesh

Bhutan

Bolivia

Cabo Verde

Cape Verde

Cameroon

Republic of Congo

Congo

Cote d'Ivoire

Ivory Coast

Djibouti

Arab Republic of Egypt

Egypt

El Salvador

Georgia

Ghana

Guatemala

Guyana

Honduras

India

Indonesia

Kenya

Kiribati

Kosovo

Kyrgyz Republic

Kyrgyz

Lao PDR

Laos

Lesotho

Mauritania

Federated States of Micronesia

Micronesia

Moldova

Morocco

Myanmar

Nicaragua

Nigeria

Pakistan

Papua New Guinea

Philippines

Samoa

Sao Tome and Principe

Sao Tome

Principe

Senegal

Solomon Islands

Sri Lanka

Swaziland

Syrian Arab Republic

Syria

Tajikistan

Timor-Leste

Ukraine

Uzbekistan

Vanuatu

Vietnam

West Bank

Gaza

Republic of Yemen

Yemen

Zambia

Afghanistan

Benin

Burkina Faso

Burundi

Cambodia

Central African Republic

Chad

Comoros

Congo

Democratic Republic of Congo

Eritrea

Ethiopia

Gambia

Guinea

Guinea-Bisau

Guinea-Bissau

Haiti

Korea

Democratic People's Republic of Korea

Liberia

Madagascar

Malawi

Mali

Mozambique

Nepal

Niger

Rwanda

Sierra Leone

Somalia

South Sudan

Sudan

Tanzania

Togo

Uganda

Zimbabwe

Variations in spelling and plurality were also included

**Filters/Limits:**

Child: birth-18 years

**Example of Database Search (PubMed):**

#1 Search (((PEWS OR "early warning" OR itat OR "critical deterioration" OR (deteriorat* AND ("severity of illness index"[mesh] OR "severity of illness" OR "trigger system" OR "trigger systems" OR "alert criteria")) AND (pediatric OR paediatric OR child OR children OR childhood OR infant* OR infancy OR adolescen*))) Filters: Publication date from 2016/11/01 to 2018/12/

#2 Search (((("developing countries"[mesh] OR "developing countries" OR "developing country" OR "developing world" OR "resource-limited" OR "resource limited" OR "limited resources" OR "resource-poor" OR "resource poor" OR "resource limiting" OR "resource-limiting" OR "resource constraine" OR "low and middle income countries" OR LMIC OR asia OR africa OR "south america" OR oceania OR "latin america" OR "global health")) OR (Albania OR Algeria OR "American Samoa" OR Samoa OR Angola OR Azerbaijan OR Belarus OR Belize OR "Bosnia and Herzegovina" OR Bosnia OR Herzegovina OR Botswana OR Brazil OR Bulgaria OR China OR Colombia OR "Costa Rica" OR Cuba OR Dominica OR "Dominican Republic " OR Ecuador OR Fiji OR Gabon OR Grenada OR "Islamic Republic of Iran" OR Iran OR Iraq OR Jamaica OR Jordan OR Kazakhstan OR Lebanon OR Libya OR "Republic of Macedonia" OR Macedonia OR Malaysia OR Maldives OR "Marshall Islands" OR Mauritius OR Mexico OR Mongolia OR Montenegro OR Namibia OR Palau OR Panama OR Paraguay OR Peru OR Romania OR Serbia OR "South Africa" OR "St. Lucia" OR "Saint Lucia" OR "St. Vincent and the Grenadines" OR "Saint Vincent" OR Grenadines OR Suriname OR Thailand OR Tonga OR Tunisia OR Turkey OR Turkmenistan OR Tuvalu)) OR (Armenia OR Bangladesh OR Bhutan OR Bolivia OR "Cabo Verde" OR "Cape Verde" OR Cameroon OR "Republic of Congo" OR Congo OR "Cote d'Ivoire" OR "Ivory Coast" OR Djibouti OR "Arab Republic of Egypt" OR Egypt OR "El Salvador" OR Georgia OR Ghana OR Guatemala OR Guyana OR Honduras OR India OR Indonesia OR Kenya OR Kiribati OR Kosovo OR "Kyrgyz Republic" OR Kyrgyz OR "Lao PDR" OR Laos OR Lesotho OR Mauritania OR "Federated States of Micronesia" OR Micronesia OR Moldova OR Morocco OR Myanmar OR Nicaragua OR Nigeria OR Pakistan OR "Papua New Guinea" OR Philippines OR Samoa OR "Sao Tome and Principe" OR "Sao Tome" OR Principe OR Senegal OR "Solomon Islands" OR "Sri Lanka" OR Sudan OR Swaziland OR "Syrian Arab Republic" OR Syria OR Tajikistan OR "Timor-Leste" OR Ukraine OR Uzbekistan OR Vanuatu OR Vietnam OR "West Bank" OR Gaza OR "Republic of Yemen" OR Yemen OR Zambia)) OR (Afghanistan OR Benin OR "Burkina Faso" OR Burundi OR Cambodia OR "Central African Republic" OR Chad OR Comoros OR Congo OR "Democratic Republic of Congo" OR Eritrea OR Ethiopia OR Gambia OR Guinea OR "Guinea-Bisau" OR "Guinea-Bissau" OR Haiti OR Korea OR "Democratic People's Republic of Korea" OR Liberia OR Madagascar OR Malawi OR Mali OR Mozambique OR Nepal OR Niger OR Rwanda OR "Sierra Leone" OR Somalia OR "South Sudan" OR Sudan OR Tanzania OR Togo OR Uganda OR Zimbabwe)

#3 Search (#1 AND #2)

#4 Search ((PEWS OR "early warning" OR itat OR "critical deterioration" OR (deteriorat* AND ("severity of illness index"[mesh] OR "severity of illness" OR "trigger system" OR "trigger systems" OR "alert criteria")))) AND #2 Filters: Child: birth-18 years

#3 OR #4
